# Targeting Toll-like receptor 4 prevents cobalt-mediated inflammation

**DOI:** 10.18632/oncotarget.7105

**Published:** 2016-01-31

**Authors:** Helen Lawrence, Amy Elizabeth Mawdesley, James Patrick Holland, John Andrew Kirby, David John Deehan, Alison Jane Tyson-Capper

**Affiliations:** ^1^ Institute of Cellular Medicine, Newcastle University, Newcastle upon Tyne, UK; ^2^ Musculoskeletal Services, Freeman Hospital, Newcastle upon Tyne, UK

**Keywords:** cobalt, metal-on-metal, antibody, TLR4, inflammation, Immunology and Microbiology Section, Immune response, Immunity

## Abstract

Cobalt-chrome alloy is a widely used biomaterial in joint replacements, dental implants and spinal rods. Although it is an effective and biocompatible material, adverse reactions to metal debris (ARMD) have arisen in a minority of patients, particularly in those with metal-on-metal bearing hip replacements. There is currently no treatment for ARMD and once progressive, early revision surgery of the implant is necessary. Therapeutic agents to prevent, halt or reverse ARMD would therefore be advantageous.

Cobalt ions activate Toll-like receptor 4 (TLR4), an innate immune receptor responsible for inflammatory responses to bacterial lipopolysaccharide (LPS) resulting in the production of pro-inflammatory cytokines and chemokines. We hypothesised that anti-TLR4 neutralising antibodies, reported to inhibit TLR4-mediated inflammation, could prevent the inflammatory response to cobalt ions in an *in vitro* macrophagecell culture model.

This study shows that a monoclonal anti-TLR4 antibody inhibited cobalt-mediated increases in pro-inflammatory *IL8, CCL20* and *IL1A* expression, as well as IL-8 secretion. In contrast, a polyclonal antibody did not prevent the effect of cobalt ions on either IL-8 or *IL1A* expression, although it did have a small effect on the *CCL20* response. Interestingly, both antibodies inhibited cobalt-mediated neutrophil migration although the greater effect was observed with the monoclonal antibody.

In summary our data shows that a monoclonal anti-TLR4 antibody can inhibit cobalt-mediated inflammatory responses while a polyclonal antibody only inhibits the effect of specific cytokines. Anti-TLR4 antibodies have therapeutic potential in ARMD although careful antibody design is required to ensure that the LPS response is preserved.

## INTRODUCTION

Cobalt-chrome alloy is widely used as a biomaterial in orthopaedic joint replacements, spinal reconstructive rods and dental implants. Over recent years concerns have emerged regarding early failure in hip replacements with cobalt-chrome metal-on-metal (MoM) bearings. These failing joint replacements are associated with high levels of cobalt and chromium in the blood and within the joint space itself. Clinical reactions include abnormal fluid collections, metallosis, muscle and soft tissue necrosis, pseudotumours, osteolysis and implant loosening [1]. These tissue responses are collectively termed ‘adverse reactions to metal debris' (ARMD) [2]. Analysis of the MoM peri-implant tissues reveals a cellular infiltrate dominated by macrophages [3] and lymphocytes [4] along with high levels of cytokines and chemokines which strongly suggests an inflammatory response.

Currently MoM total hip replacement revision rates range from 15% to 35% at 7 years compared to 2% for conventional metal-on-polyethylene devices [5]. Despite the high rate of revision for MoM hip replacements there are still more than 1 million patients worldwide with these hips implanted and un-revised [6], which require ongoing surveillance. The major therapeutic option for pseudotumours and ARMD is currently removal of all cobalt-chrome alloy and revision to a ceramic or polyethylene hip implant. This is a more complex surgical procedure than primary hip replacements due to the erosion bone stock and loss of muscle and soft tissue, and post-revision recovery is often slow and incomplete. Hip revision for pseudotumours has been associated with operative risks comprising 75% blood transfusion, 19% recurrent dislocation, 19% nerve palsy, 13% component loosening and 40% risk of re-revision [7, 8] causing distress to patients and a considerable financial burden to the NHS. An adjuvant therapeutic agent that could ameliorate or inhibit the initiation of the inflammatory signalling cascade that results in ARMD and thus lessen the surgical insult or even dampen down ARMD without the need for surgical intervention would therefore be attractive. Current research into ARMD therapies focuses on modulation of the ongoing inflammatory response by polarising macrophages towards an anti-inflammatory phenotype [9] or inhibiting the function of pro-inflammatory cytokines such as CCL2 [10] and have shown positive results in *in vivo* models. However they are made more complex by the pleiotropic and redundant nature of cytokines and chemokines.

Recent studies by our group and others have shown that cobalt ions generated by MoM hip articulation can activate the innate immune receptor Toll-like receptor 4 (TLR4) [11-14] which is most commonly known as the receptor for Gram negative bacterial lipopolysaccharide (LPS). Cobalt-mediated TLR4 activation increases the secretion of pro-inflammatory cytokines such as interleukin-8 (IL-8, chemokine (C-X-C motif) ligand 8 or CXCL8), interleukin-6 (IL-6) and chemokine (C-X-C motif) ligand 10 (CXCL10) in macrophages and endothelial cells [11, 14]. A small molecule TLR4 antagonist, CLI-095, is able to prevent these responses [14] but its therapeutic use is limited because of its intracellular mechanism of action.

Antibodies represent a successful and rapidly evolving therapeutic strategy. Both monoclonal and polyclonal anti-TLR4 antibodies are currently under investigation as therapeutic agents in the prevention of septic shock induced by LPS [15], and a number of anti-TLR4 antibodies are currently in clinical trials to improve test their effectiveness in diseases including rheumatoid arthritis [16]. We therefore hypothesised that anti-TLR4 neutralising antibodies could prevent cobalt-mediated TLR4 activation, using changes in cytokine secretion and expression by macrophages as markers of receptor activation and inhibition.

## RESULTS

### Antibody cytotoxicity

**Figure 1:**
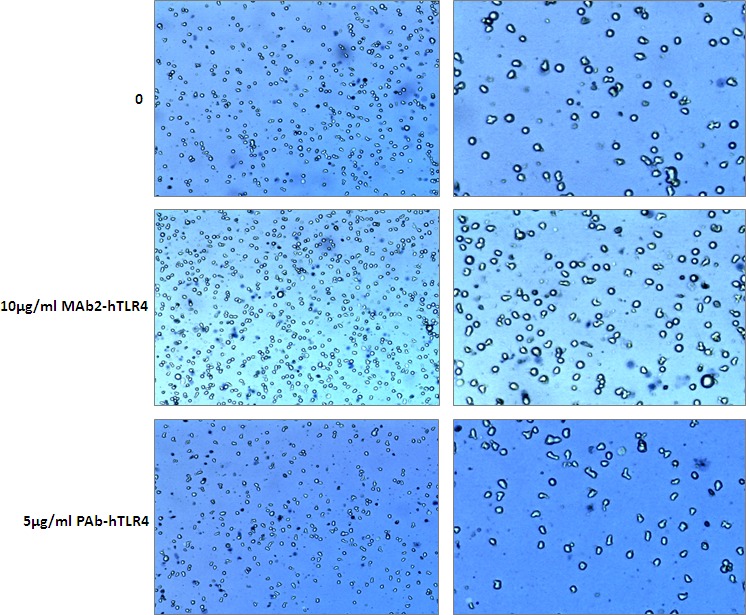
Cytotoxicity assay for MAb2-hTLR4 and PAb-hTLR4 MonoMac 6 cells were incubated with 10μg/ml MAb2-hTLR4 or 5μg/ml PAb-hTLR4 for 16h. A cytotoxicity assay was conducted using trypan blue staining and cell counting on a Luna II automated cell counter. Cell viability was 100% in untreated and MAb2-hTLR4-treated cells, and 94% in those incubated with PAb-hTLR4. Images on the right are magnified versions of those on the left.

MonoMac 6 cells were incubated with 10μg/ml MAb2-hTLR4 or 5μg/ml PAb-hTLR4 for 16h. Cytotoxicity was assessed by trypan blue staining and cells were counted using a Luna II automated cell counter. MAb2-hTLR4 did not affect MonoMac 6 cells viability (Figure 1) while PAb-hTLR4 decreased cell viability from 100% to 94% (Figure 1).

### Inhibition of cobalt-mediated inflammatory responses by a monoclonal anti-TLR4 antibody

**Figure 2:**
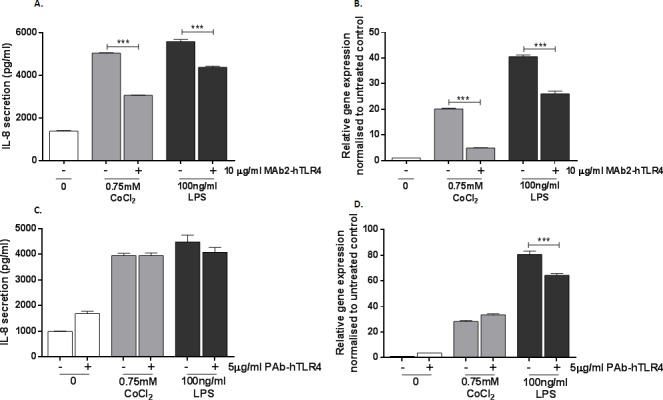
Effect of monoclonal and polyclonal anti-TLR4 neutralising antibody on cobalt-mediated IL-8 expression and secretion **A.** MonoMac 6 cells were pre-incubated with 10μg/ml MAb2-hTLR4 for 1h and then stimulated with 0.75mM CoCl_2_ or 100ng/ml LPS for 16h. Supernatant was collected and IL-8 secretion was measured by ELISA. **B.** qRT-PCR was performed to evaluate the effect of MAb2-hTLR4 on *IL8* expression. **C.** MonoMac 6 cells were pre-incubated with 5μg/ml PAb-hTLR4 for 10 minutes followed by stimulation with 0.75mM CoCl_2_ or 100ng/ml LPS for 16h. Supernatant was collected and IL-8 secretion measured by ELISA. **D.** qRT-PCR was performed to evaluate the effect of PAb-hTLR4 on *IL8* expression. Data is representative of three independent experiments and statistical significance was calculated by one-way analysis of variance (ANOVA) with Tukey's test for multiple comparisons comparing all samples to each other.

TLR4-expressing MonoMac 6 cells have previously been shown to upregulate IL-8 secretion and expression when challenged with cobalt ions [14] and were therefore selected as an *in vitro* cell model for this study, using IL-8 as a marker of TLR4 activation. MonoMac 6 cells were pre-treated with 10μg/ml MAb2-hTLR4 for 1h prior to 16h stimulation with 0.75mM CoCl_2_ or 100ng/ml LPS. IL-8 protein levels were measured by ELISA and *IL8* expression was quantified by qRT-PCR. CoCl_2_ and LPS both significantly increased IL-8 secretion to approximately 5000pg/ml (p<0.001) (Figure 2A). Pre-treatment with MAb2-hTLR4 significantly decreased IL-8 secretion to 3000pg/ml in CoCl2-stimulated cells (*p*<0.001) and 4000pg/ml in LPS-stimulated cells. *IL8* gene expression followed a similar pattern with significant upregulation in expression following CoCl_2_ and LPS stimulation (both *p*<0.001) (Figure 2B). MAb2-hTLR4 significantly inhibited CoCl_2_-mediated *IL8* expression, reducing it from 20-fold to 5-fold (*p*<0001). The monoclonal antibody also prevented the *IL8* expression increase induced by LPS (*p*<0.001).

### A polyclonal anti-TLR4 antibody does not prevent cobalt-mediated IL-8 changes

A polyclonal anti-TLR4 neutralising antibody, PAb-hTLR4, is reported to inhibit inflammatory responses to TLR4 ligands. Using IL-8 expression and secretion by MonoMac 6 cells as a marker of inflammation, the ability of PAb-hTLR4 to prevent the cellular response to cobalt was assessed. MonoMac 6 cells were pre-treated with 5μg/ml PAb-hTLR4 for 10 minutes prior to 16h stimulation with either 0.75mM CoCl_2_ or 100ng/ml LPS. IL-8 secretion was measured by ELISA and expression by qRT-PCR.

PAb-hTLR4 did not inhibit CoCl_2_ or LPS-mediated IL-8 secretion (Figure 2C) (*p*>0.4 in both cases). In untreated cells there was an increase in IL-8 secretion in the presence of the antibody although this did not reach statistical significance (*p*=0.051). *IL8* expression following LPS stimulation was inhibited by PAb-hTLR4 (*p*<0.001) (Figure 2D). However the antibody could not prevent the effect of cobalt ions on *IL8* expression (*p*=0.095).

### MAb2-hTLR4 prevents cobalt-mediated expression of *CCL20* and *IL1A*

**Figure 3:**
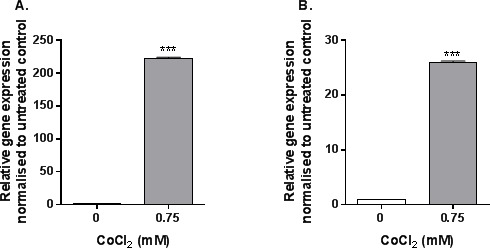
*CCL20* and *IL1A* expression following CoCl_2_ stimulation MonoMac 6 cells were stimulated with 0.75mM CoCl_2_ for 4h before qRT-PCR was conducted to assess A. *CCL20* and B. *IL1A e*xpression. Data is representative of three independent experiments and statistical significance was calculated by Student's *t* test.

Using qRT-PCR arrays 4h stimulation of MonoMac 6 cells with 0.75mM CoCl_2_ was found to upregulate expression of pro-inflammatory *CCL20* and *IL1A*. The array results were subsequently validated by TaqMan-based qRT-PCR. This analysis confirmed a significant upregulation in both *CCL20* (Figure 3A) and *IL1A* (Figure 3B) expression following 4h exposure of MonoMac 6 cells to 0.75mM CoCl_2_ (both *p*<0.001), showing the potential of these genes as targets for studying antibody efficacy.

**Figure 4:**
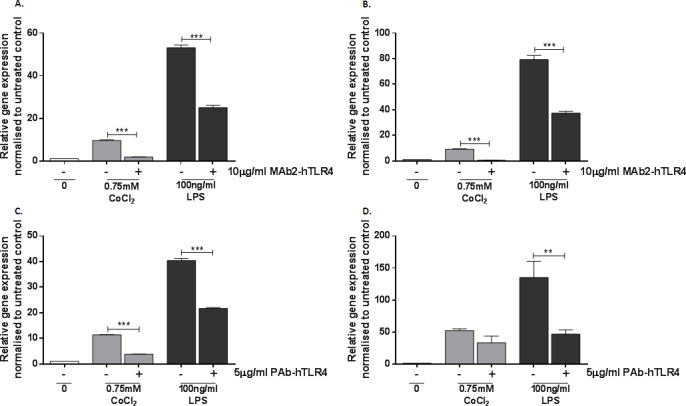
Effect of MAb2-hTLR4 and PAb-hTLR4 on cobalt-mediated *CCL20* and *IL1A* expression **A.** & **B.** MonoMac 6 cells were pre-treated with 10μg/ml MAb2-hTLR4 for 1 hour prior to 16h stimulation with 0.75mM CoCl_2_ or 100ng/ml LPS. RNA was isolated and cDNA synthesised by reverse transcription. *CCL20* (A) and *IL1A* (B) expression was quantified by qRT-PCR. C&D. MonoMac 6 cells were pre-treated with 5μg/ml PAb-hTLR4 for 10 minutes before 16h stimulation with 0.75mM CoCl_2_ or 100ng/ml LPS. RNA was isolated and cDNA synthesised by reverse transcription. *CCL20* **C.** and *IL1A* **D.** expression was quantified by qRT-PCR. Data is representative of three independent experiments and statistical significance was calculated by one-way ANOVA with Tukey's test for multiple comparisons comparing all samples to each other.

The ability of MAb2-hTLR4 to inhibit cobalt-mediated changes in *CCL20* and *IL1A* expression was therefore investigated using the same treatment protocol as described for the IL-8 assays. *CCL20* expression was upregulated approximately 10-fold by CoCl_2_ stimulation (*p*<0.001) whilst the effect of LPS was greater, with a 50-fold increase in expression (*p*<0.001) (Figure 4A). MAb2-hTLR4 significantly decreased expression of CCL20 in response to CoCl2 and the positive control LPS (*p*<0.001 in both cases). *IL1A* expression was also significantly increased by CoCl_2_ (*p*=0.0283) and LPS (*p*<0.001) (Figure 4B). In the presence of MAb2-hTLR4 CoCl_2_-mediated IL1A expression was significantly decreased (*p*=0.0224) as was that of LPS (*p*<0.001).

### A polyclonal anti-TLR4 antibody can inhibit other cobalt-mediated inflammatory effects

Expression of *CCL20* and *IL1A* was investigated to validate the inability of PAb-hTLR4 to inhibit TLR4 activation by cobalt ions using the same treatment protocol described for the IL-8 assays. *CCL20* expression was assessed by qRT-PCR and found to be abrogated by the polyclonal antibody (Figure 4C) (*p*<0.001) but there was no effect on *IL1A* (Figure 4D) (*p*=0.8246). PAb-hTLR4 inhibited expression of both genes in response to LPS stimulation (*p*<0.001 for *CCL20* and p=0.0044 for *IL1A*).

### Cobalt-mediated neutrophil migration is inhibited by TLR4 neutralising antibodies

Inflammatory cell infiltration is a hallmark of MoM peri-implant tissues. As IL-8 secretion is significantly increased by cobalt ions and IL-8 is chemotactic for neutrophils, the effect of cobalt and the neutralising antibodies on primary neutrophil migration was assessed. MonoMac 6 cells were pre-stimulated with MAb2-hTLR4 or PAb-hTLR4 as described earlier before 16h stimulation with 0.75mM CoCl_2_ or 100ng/ml LPS. Conditioned media was collected and used in a transwell neutrophil migration assay.

**Figure 5:**
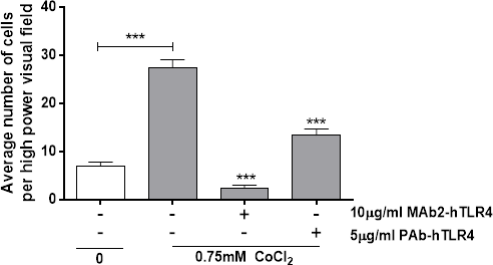
Anti-TLR4 neutralising antibodies inhibit cobalt-mediated neutrophil chemotaxis MonoMac 6 cells were pre-treated with 10μg/ml MAb2-hTLR4 for 1 hour or 5μg/ml PAb-hTLR4 for 10 minutes before 16h stimulation with 0.75mM CoCl_2_ or 100ng/ml LPS. Supernatant was collected and chemotactic properties assessed using a transwell neutrophil migration assay. Data is representative of three independent experiments and statistical significance was calculated by one-way ANOVA with Tukey's test for multiple comparisons comparing all samples to each other.

Conditioned media from cobalt-stimulated MonoMac 6 cells significantly increased neutrophil migration (*p*<0.001) (Figure 5). Both MAb2-hTLR4 and PAb-hTLR4 pre-incubation significantly inhibited this effect (both *p*<0.001) although the monoclonal antibody induced a greater reduction in migration than the polyclonal.

## DISCUSSION

This study demonstrates that a monoclonal anti-TLR4 neutralising antibody (MAb2-hTLR4) inhibits pro-inflammatory *IL8, IL1A* and *CCL20* expression and IL-8 secretion by MonoMac 6 cells following cobalt stimulation. MAb2-hTLR4 provided more effective inhibition than the polyclonal antibody PAb-hTLR4 which did not prevent cobalt-mediated IL-8 or *IL1A* responses although it did inhibit *CCL20* expression. Despite the differences in their inhibitory effect both neutralising antibodies prevented neutrophil migration in response to cobalt stimulation, with a greater effect observed for the monoclonal antibody.

The differences in inhibition between the monoclonal and polyclonal antibodies are likely due to the fact that cobalt and LPS bind to TLR4 in different ways; cobalt binds directly to the receptor within a ‘histidine pocket' composed of histidine residues H431, 456 and 458 [11] while LPS initially binds LPS binding protein (LBP) before transfer across a series of adaptor proteins to the receptor itself. If an antibody blocks an adaptor protein binding site then it may inhibit LPS without having an effect on the cobalt response. However as PAb-hTLR4 blocked *CCL20* expression it may have a small effect on receptor homodimerisation which is required for activation by both LPS and cobalt ions [11].

The differences in cobalt and LPS binding sites could be exploited for the development of an antibody to prevent ARMD. For an anti-TLR4 antibody to be successful in preventing the inflammatory response to cobalt ions it is essential that the immunological response to LPS is maintained or else there is a high risk of overwhelming bacterial infection and potentially lethal septic shock. The development of a novel antibody targeting the cobalt-specific binding site of TLR4 could prevent activation by cobalt ions while still allowing an inflammatory response to LPS.

This study focused on a select group of pro-inflammatory cytokines and chemokines induced by cobalt based on previous work performed by the group. However individual cytokine targets are of limited value when investigating the potential of a therapeutic agent as it is the cumulative effect of cytokines and chemokines that leads to ARMD. For this reason neutrophil migration assays were performed to assess the capacity of the antibodies to prevent cobalt-mediated neutrophil recruitment. Both antibodies inhibited neutrophil migration; the effect of the monoclonal antibody was greater than that of the polyclonal which supports the other data described in this study. It also indicates that neutrophil chemokines other than IL-8 are inhibited by PAb-hTLR4. Additional *in vitro* functional assays or in an *in vivo* mouse model with humanised TLR4 could be used to further investigate these effects and antibody efficacy.

In summary, our data shows that anti-TLR4 neutralising antibodies can prevent cobalt activation of the receptor and subsequent inflammatory cytokine upregulation by human macrophages. The design of an antibody for ARMD therapy will be critical to provide the most effective inhibition of cobalt-mediated inflammation while still ensuring that the LPS-binding capacity of TLR4 is preserved. This work suggests that TLR4 has excellent potential as a therapeutic target in the prevention of ARMD.

## MATERIALS AND METHODS

### MonoMac 6 cells

MonoMac 6 cells are a TLR4-expressing human macrophage cell line derived from acute monocytic leukaemia. MonoMac 6 cells were cultured in complete RPMI-1640 medium supplemented with 10% foetal bovine serum (FBS), 2mM L-glutamine, 50 U/ml penicillin and 50μg/ml streptomycin (all Sigma-Aldrich, Gillingham, UK).

### Metal ion stimulation

Cobalt chloride hexahydrate (CoCl_2_) was diluted in complete cell culture medium and used at 0.75mM, a physiological concentration optimised in a previous study [14]. TLR4-specific LPS (from *E. coli* serotype J5, Alexis Biochemicals, San Diego, USA) was used at 100ng/ml as a positive control for receptor activation.

### Neutralising antibodies

PAb-hTLR4 (Invivogen, San Diego, USA) is a polyclonal rat IgG antibody reported to neutralise TLR4 and prevent activation by its ligands [17]. MAb2-hTLR4 (Invivogen) is a monoclonal mouse IgG1 antibody (clone 3C3) reported to neutralise TLR4 and prevent receptor activation by agonists [15].

### Cytotoxicity

Cytotoxicity was assessed by trypan blue staining; 10μl trypan blue dye (Logos Biosystems, Anyang-City, South Korea) was mixed with 10μl cell suspension and the number of live and dead cells was counted using a Luna-II Automated Cell Counter (Logos Biosystems). Data was normalised to 100% viability for untreated cells.

### Quantitative real-time PCR (qRT-PCR)

RNA was isolated using a Qiagen RNeasy Mini kit (Qiagen, Venlo, Netherlands) and cDNA synthesized using Superscript III reverse transcriptase (ThermoFisher Scientific, Massachusetts, USA). qRT-PCR was performed using TaqMan gene expression probes (ThermoFisher Scientific). Each reaction contained 5μl TaqMan Gene Expression Mastermix (ThermoFisher Scientific), 8μl diluted cDNA, 2.5μl nuclease-free H_2_O and 0.5μl TaqMan Gene Expression Assay. No-template controls containing 8μl H_2_O instead of cDNA were included for all assays. All target genes were normalised to *GAPDH* as a housekeeping gene. Reactions were performed in triplicate.

### ELISA

Enzyme-linked immunosorbent assays (ELISA) were used to quantify cytokine secretion by MonoMac 6 cells. IL-8 secretion was quantified using a Human IL-8 ELISA kit (Peprotech, London, UK) as described previously [14].

### Transwell neutrophil migration assay

Neutrophils were isolated from the whole blood of healthy volunteers by dextran sedimentation (Dextran T500, Pharmacosmos, Holbaek, Denmark) and centrifugation on Percoll (GE Healthcare, Buckinghamshire, UK) density gradients as previously described [18].

A 24-well companion plate (VWR International, Pennsylvania, USA) was blocked with 1% bovine serum albumin (BSA) (Sigma Aldrich) for 1 hour after which 800μl conditioned media from stimulated MonoMac 6 cells was added to each well. A cell culture insert with 3μm pore filter (VWR International) was placed in each well and 500,000 neutrophils added to the upper chamber of each insert. The plate was incubated at 37°C for 2 hours to allow for cell migration. Excess media was removed and filters incubated overnight in 100% methanol. Filters were stained with haematoxylin for 30 minutes and washed in Scott's tap water for 10 minutes. This was followed by dehydration in increasing ethanol concentrations (50, 75, 90 and 100%) for 2 minutes each. Filters were air-dried for a minimum of three hours and were excised and mounted using DPX mountant. Adhered neutrophils were counted at x40 magnification.

### Statistical analysis

Statistical analysis was performed using GraphPad Prism 6.0 (GraphPad Software Inc., San Diego, USA). All error bars show the standard error of the mean (SEM) unless otherwise stated. The analysis method is described for each individual experiment. Statistical significance is shown as follows *=p<0.05, **=p<0.01, ***=p<0.001.
